# The Economical Management of an Efficient Voluntary Hospital

**Published:** 1905-04-01

**Authors:** 


### HOSPITAL ADMINISTRATION.

### CONSTRUCTION AND ECONOMICS.

### THE ECONOMICAL MANAGEMENT OF AN EFFICIENT VOLUNTARY HOSPITAL.

The following are the conditions to be observed by competitors for Mr. Edgar Speyer's prize of £100 and a silver cup for the best essay on the above subject:—

1. The Competition is open to the paid secretaries and assistant secretaries of all voluntary hospitals in England, Scotland, and Ireland. The expressions "secretary" and "assistant secretary" include the persons who perform the ordinary secretarial duties and the persons who, in their absence, perform those duties, provided that both are paid officers of the institution. In cases of doubt it will be better to state the circumstances for approval beforehand.
2. The essays must be type-written on one side of the paper only.
3. No limit is placed on the length of the essays. Every essay must conclude with a summary of its contents. In cases of equality the preference will be given to the most concise paper.
4. The name of the author must not be written on the essay, but must be enclosed in a sealed envelope endorsed with a motto, a copy of which must also be typed on the essay.
5. The unsuccessful essays and the sealed envelopes accompanying them will be destroyed. The latter will not be opened.
6. Each sealed envelope must contain the accompanying letter, signed by the author of the essay.
7. Mr. Edgar Speyer reserves to himself the right to decide, in consultation with such gentlemen as he may invite to confer with him, on the merits of the essays.
8. Essays must be delivered, addressed to the Honorary Secretaries of King Edward's Hospital Fund, 81 Cheapside, E.C., and marked on the envelope "Essay," not later than Friday, September 15th, 1905.
9. Inquiries should be addressed to the Honorary Secretaries of King Edward's Hospital Fund, 81 Cheapside, E.C.

The letter referred to in condition 6 provides that the competitor who is successful in winning the prize must consent to its publication, free of expense to himself, under his own name, without further payment. The successful competitor will have further to agree to make over the copyright if called upon to do so to be used for the benefit of King Edward's Hospital Fund for London. The Prince of Wales has consented to present the prize to the successful competitor.

We can see very good reasons of an administrative character, having regard to the criticisms which a man of knowledge must in all honesty make in writing on hospital economy, which in our judgment should lead Mr. Edgar Speyer and those immediately concerned to waive the enforcement of the condition that the essay when printed shall bear upon its face the name of its author.

